# The Usefulness of Brief Family Health Conversations Offered to Families Following the Diagnosis of Breast Cancer

**DOI:** 10.1177/1074840720966759

**Published:** 2020-10-28

**Authors:** Annette Holst-Hansson, Vedrana Vejzovic, Ewa Idvall, Anne Wennick

**Affiliations:** 1 Lund University, Sweden; 2 Malmö University, Sweden

**Keywords:** breast cancer, family nursing intervention, Family Health Conversations, qualitative research

## Abstract

Currently, there are few studies which examine targeted family-focused support when a family member is diagnosed with breast cancer. Thus, the aim of this study was to explore families’ experiences of participating in a family nursing intervention identified as Brief Family Health Conversations (BFamHC) following the diagnosis of breast cancer. Semi-structured family interviews were conducted with nine families (including 29 family members) 2 weeks following the family-focused intervention of three sessions of BFamHC. Thematic analysis was used to analyze the data. Families reported the BFamHC as positive and as a unique kind of family health conversation, one that afforded them the opportunity to communicate and share their experiences as a family group. A family conversation, even one as time-limited as BFamHC, offered a sense of relational sharing and togetherness, thus preventing feelings of isolation and vulnerability. Therapeutic family-focused conversations, such as BFamHC, hold promise as a useful family nursing intervention following the diagnosis of breast cancer.

Breast cancer is an illness that not only affects the individual but also the family as a unit and its individual members (Coyne et al., 2012; Dieperink et al., 2017; Holst-Hansson, 2018; Holst-Hansson et al., 2017, 2018; Möllerberg et al., 2020). Family Systems Nursing (FSN) is an overarching theoretical orientation that acknowledges and addresses the multiple systems involved in the experience of health and illness: the individual, the family, the health care provider(s), and larger systems—all of whom are pivotal to health and healing (Bell, 2009; Bell & Wright, 2015; Leahey & Svavarsdottir, 2009; Shajani & Snell, 2019; Wright & Leahey, 2013). More specifically, FSN involves family members in therapeutic conversations about the impact of the diagnosis of illness (e.g., breast cancer) and treatment on the family unit and their experiences in the health care system (Leahey & Svavarsdottir, 2009; Wright & Leahey, 2013). FSN interventions are offered through therapeutic conversations that have been found to be useful, particularly when a family member is diagnosed with a serious and/or long-term illness (Bell, 2016; Petursdottir & Svavarsdottir, 2019; Svavarsdottir et al., 2020). This study explored families’ experiences of participating in therapeutic family nursing conversations led by an oncology nurse following a diagnosis of breast cancer.

## Literature Review

To support families following a breast cancer diagnosis and to understand their experiences of illness, the conceptual systemic lens known as FSN, as described by Wright and Leahey (2013), may be beneficial. The rationale for FSN is that it addresses interaction, relationships, and reciprocity between multiple systems levels—including the diagnosis, the person with the diagnosis, the family, and the health care professionals, as well as the health care system and society (Wright & Bell, 2009). Furthermore, it is a systemic approach that focuses on the interactions and relationships within the family and larger systems. It aims to preserve health and facilitate healing, with the family members’ experiences and beliefs seen as equally significant as those of the person with the diagnosis (Wright & Leahey, 2013). Yet, when it comes to intervention studies on families stricken by parental cancer, only a small number of family members are included (Inhestern et al., 2016).

Existing systematic reviews focused on families experiencing cancer offer an overview of structured interventions targeting children coping with their parent’s cancer (Niemelä et al., 2010) and families of persons receiving palliative care (Kühne et al., 2012). The third, and most recent, of these reviews offers an overview of existing interventions and support programs from 36 studies that identify barriers and facilitators of psychosocial interventions for families affected by parental cancer (Inhestern et al., 2016). However, what these reviews have in common is that they include all types of parental cancer, which makes it hard to claim those that might be beneficial for families following the specific diagnosis of breast cancer. Consequently, there is a need to explore how families’ needs are best supported by family nursing intervention following a breast cancer diagnosis. The existing research recommends that interventions should be flexible, easy to access, and offered as routine care to reach a broader range of families in need of support.

FSN is based on the theoretical orientation of the Calgary Family Assessment Model (CFAM), the Calgary Family Intervention Model (CFIM; Wright & Leahey, 2013), and the Illness Belief Model (IBM; Wright & Bell, 2009). A family intervention that has been culturally adapted to Sweden from the practice models of FSN is called Family Health Conversations (FamHC; Benzein et al., 2008, 2017). FamHC are therapeutic conversations offered to families to create a context for change in family health and support the creation of new beliefs, meanings, and relationship opportunities specific to the illness story narrated by the family (Benzein et al., 2008). The conversations have a family focus and begin with the family’s stories of their illness experience (Östlund et al., 2016). FamHC are often held in a series of three conversations that last about 1 hr each (Benzein et al., 2008; Östlund et al., 2016).

As this study took place in Sweden, and because Benzein and colleagues (2008, 2017) have culturally adapted FamHC to a Swedish context, it was considered a natural choice for the present study. Those invited to participate in this study were families of women diagnosed with breast cancer and receiving curative radiation therapy (RT). As a treatment, RT is generally given once a day, 5 days a week, with a total number of treatments ranging from 15 to 33 days, with each session taking about 10 to 20 min. This was taken into consideration when choosing between the full-length and shorter conversation models. We choose the latter model, Brief Family Health Conversations (BFamHC), because we wanted the time frame for the conversations to coincide with the treatment time to some extent. The end of active treatment, which for women with breast cancer is usually RT, is often associated with a decline in support from health care professionals (Hewitt & Ganz, 2006). For that reason, we decided to schedule the BFamHC during the end of active treatment. Moreover, it was especially important to do so considering that family needs at this time might not be assessed or dealt with later.

## Rationale for This Study

In response to the aforementioned shortcomings in current studies concerning the targeted support and specific needs of relatives of a family member diagnosed with breast cancer, this study explored the experiences of family members participating in therapeutic conversations. Previous studies have shown that parents have difficulties committing to regular therapeutic sessions due to time constraints (Inhestern et al., 2016; Thastum et al., 2006), therefore the less time-consuming BFamHC was chosen for these conversations. Family health conversations have been used previously with families experiencing various chronic illnesses (Benzein et al., 2015; Dorell et al., 2016; Robinson & Wright, 1995; Östlund et al., 2016; Voltelen et al., 2016). However, to our knowledge, they have not yet been used to explore the families’ experiences following a breast cancer diagnosis.

## Aim

The aim of the study was to explore families’ experiences of participating in BFamHC following a breast cancer diagnosis.

## Method

### Design

This was a qualitative inductive study involving interviews with families after they had participated in BFamHC following a breast cancer diagnosis. The analytical approach chosen was thematic analysis, which has been recognized by Braun and Clarke (2006) as a suitable method for elucidatory studies aiming at identifying, analyzing, and reporting patterns, as well as making interpretations of the data (Boyatzis, 1998). The participants in this study participated in three BFamHC sessions during the outpatient RT period following the diagnosis of breast cancer of the mother of the family. Benzein and colleagues (2008) recommend that each family is offered three conversations when conducting BFamHC.

### Description of the Family Nursing Intervention: BFamHC Offered During the Radiotherapy Period

In the first BFamHC, all family members were invited to narrate their experience of their present situation and to listen to each other’s stories. The first author (A.H.H.), who is a registered nurse with extensive clinical experience of oncology and breast cancer care, theoretical knowledge of FSN, and experience in offering family health conversations (FamHC), conducted the BFamHC in the role of conversational leader. Reflective, interventive questions (Benzein et al., 2008; Wright & Bell, 2009; Wright & Leahey, 2013) were posed during the BFamHC by the conversational leader (A.H-.H.). Example questions were as follows: “In what way are you affected as a family by X’s breast cancer?”; “Who in the family is most affected by the diagnosis?”; and “What affects how you handle the situation?” In collaboration with the conversational leader (A.H.H.), the family prioritized the most relevant topics.

The second BFamHC focused on beliefs and issues brought up in the first conversation, with the conversational leader (A.H.H.) posing questions such as “What are your reflections from the last time we met?”; “How do you experience your situation as a family right now?”; and “What is the biggest challenge for your family right now?” The third conversation revolved around the strengths and resources within the family and how these might help the family to reduce difficulties and increase well-being in the future. Consequently, the questions posed during the third conversation were as follows: “Can you mention a situation when you feel that you are in control or feel strong?”; “What do you think the future holds for your family?”; and “Is there anyone in your vicinity who gives you strength as a family?” During the BFamHC, the conversational leader (A.H.H.) sought to understand each family’s structure with the use of a genogram and also used an ecomap to visualize the family’s involvement with other individuals and larger systems. In addition, the conversational leader (A.H.H.) offered the families her reflections and commendations about individual and family strengths. The BFamHC were conducted in the participants’ home in the evenings and ranged from 15 to 40 min and lasting an average of 25 min.

### Participants

A total of nine families (including 29 family members) participated in this study (see Table 1). The inclusion criteria were as follows: Swedish-speaking families who had participated in a series of three BFamHC during the RT period; with children aged 12 and older; and where the woman (and mother) was diagnosed with breast cancer in an early stage and receiving curative RT at a university hospital in the south of Sweden. As children begin to develop their abstract thinking—implying, among other things, their ability to have their own complex opinions and perceive multidimensional situations by the age of 12 (Belmont, 1989; Piaget, 1970/1972)—the lower age limit was set at 12 years. A total of 10 families who met the study’s eligibility criteria were consecutively invited to participate; one family declined participation for personal reasons. For the participating families, a time and location for the interview, chosen by the family, was scheduled.

**Table 1.** An Overview Describing the Family Members’ Relation and Characteristics When Participating in Brief Family Health Conversations (BFamHC) (Family Members, *n* = 29).

Participants	Age of the woman diagnosed with breast cancer (years)	Relation to the woman diagnosed with breast cancer (age, in years) when participating in BFamHC	Relation to the woman diagnosed with breast cancer (age, in years) when participating in BFamHC	Relation to the woman diagnosed with breast cancer (age, in years) when participating in BFamHC
Family 1	50	Son (21)	—	—
Family 2	50	Son (13)	Son (12)	—
Family 3	56	Daughter (25)	—	—
Family 4	47	Daughter (24)	Son (22)	—
Family 5	46	Daughter (15)	Son (13)	Son (12)
Family 6	52	Husband (46)	Daughter (24)	Son (17)
Family 7	48	Husband (51)	Son (14)	—
Family 8	43	Husband (44)	Daughter (15)	Son (12)
Family 9	47	Husband (57)	Son (13)	Son (13)

The mothers in the participating families all had undergone breast cancer surgery; six of them had received chemotherapy, and they all received RT for 25 days (*n* = 6), 20 days (*n* = 2), and 15 days (*n* = 1). The time from diagnosis to the family interviews ranged from 3 to 9 months (see Table 1).

### Ethical Considerations

In accordance with the Declaration of Helsinki (World Medical Association, 2013), the families were informed that participation was voluntary and could be withdrawn at any time without any negative consequences. They were also assured that confidentiality would be respected throughout the research process. In addition, the families were given time to discuss the study and their participation at home before making their decision; moreover, they decided the time and location for the BFamHC and the interviews to minimize inconvenience in their life. Prior to data collection, written informed consent was obtained from all participants except children younger than 15 years, who gave their verbal assent and whose parents gave their written informed consent. The first author gave the families a card with contact information to a counselor at the clinic for further support if they were emotionally affected due to participating. Ethical approval was obtained from the Regional Ethical Review Board (No. 2016/649).

### Data Collection of the Research Interviews

To explore the families’ experiences of participating in BFamHC, semi-structured research interviews influenced by Eggenberger and Nelms (2007) were conducted by the second author (V.V.) 2 weeks after the last BFamHC. The rationale for conducting family research interviews is that they emphasized the shared family experience and family meanings. Furthermore, they highlighted the multiple voices within the families—voices that were both autonomous and related (Donalek, 2009; Eggenberger & Nelms, 2007; Hartrick & Lindsey, 1995). A calm, relaxed approach that focused on the family as a whole and acknowledged individual perspectives was used during the research interviews. Each research interview started with a clarification of the purpose of the interview and an open-ended question: “Can you please tell me how you experienced the BFamHC?”

An interview guide consisting of open-ended questions was used to invite family members to freely narrate their experiences of BFamHC. The questions focused on positive and negative experiences of participating in BFamHC, the possible contribution the therapeutic conversations had to any changes observed in the family, and the family’s possibility to influence the therapeutic conversations. Questions were also asked about the format and the content of the BFamHC. These research interviews were performed in the families’ homes (*n* = 9), lasted for 20 to 45 min (*md* = 29), and were electronically recorded for later transcription.

### Data Analysis of the Research Interviews

Data from the research interviews were analyzed in six stages using thematic analysis corresponding to the work of Braun and Clarke (2006). The first step was for the research team to familiarize themselves with the data; it commenced with the first author (A.H.H.) transcribing the interviews and making notations about additional interview features such as voice inflection, silence, and laughter. The first (A.H.H.) and second (V.V.) authors then read and reread all transcripts to obtain an understanding of the overall text. Thereafter, they began to identify data relevant to the research question.

The second stage in the analytic process was coding, which was done so that the codes captured both the semantic and the conceptual reading of the data. Furthermore, the authors independently coded every data item, ending this stage by collecting all codes to organize the qualitative data. In the next stage, both authors (A.H.H., V.V.) independently identified potential themes from the codes, based on patterns and clusters of meaning within the data set, while keeping the aim of the study in mind. In the fourth stage, themes were jointly reviewed and inspected to ascertain that they were accurate in relation to both the coded data and the transcripts. This stage also involved reflections on whether the themes were convincing and on the relationships between the themes. In the fifth stage, the essence of the themes was pinned down by defining and naming them. Thereafter, all authors (A.H.H., V.V., E.I., & A.W.) discussed the findings to detect potential biases or inappropriate subjectivity, and all disagreements were discussed until a consensus was achieved. The last stage entailed writing the analytic story and intertwining it with significant and representative quotes to present a comprehensible and credible story about the families’ experience of participating in BFamHC.

## Results

The families reported that participation in the BFamHC had affected them positively, albeit too late in the treatment trajectory. However, while some family members wanted additional conversations to further process insights brought forth by the BFamHC, others found three conversations to be suitable and adequate; some of the children related that two conversations would have been sufficient.

Families reported feeling seen and important. Furthermore, involvement in the BFamHC made them feel stronger as a family and more able to cope with future stressors and events. The BFamHC allowed them not only to gather and to review their journey as a family through the breast cancer trajectory but also facilitated their path back to what they described as a new version of their ordinary life as they knew it from before the illness. Although the family members had experienced both reluctance and distress prior to the first BFamHC, their initial unease was transformed into a more comfortable state during the conversations. They felt that something had happened to them during the BFamHC; however, they found it difficult to define the exact effect or impression. The opportunity to participate in the BFamHC in their homes was highly appreciated by the families, as it was experienced as more relaxed, private, and safe.

According to the family members, the BFamHC helped them to verbalize their feelings and thoughts, and it gave them an insight into each other’s emotions, fears, and questions. Families’ experiences of participating in BFamHC were identified in three key themes: bringing everything out in the open; being confirmed as an individual and as a family; and gaining an unexpected insight.

### Bringing Everything Out in the Open

The families experienced the BFamHC as a different than usual kind of conversation about their illness experiences that had afforded them an opportunity to verbalize their feelings: Furthermore, they felt that some issues would have not been brought to the surface otherwise. One son related,

We talked at home, yet not in that way. I think it was good to get the opportunity to sit down together and talk and get the others’ perspective as well, especially Mom’s and Dad’s, what they really think.

Both parents and children disclosed that they had avoided verbalizing thoughts and feelings due to wanting to be considerate of other family members and protect each other. Examples of earlier suppressed feelings and thoughts concerned not only a fear of their wife’s/mother’s death and worries about the treatments and their side-effects but also uncertainty about how to communicate such feelings and thoughts. The sharing of these feelings and thoughts now gave the family members a sense of relief. This was especially evident among the parents, who wanted to stay strong in front of their children and to protect them. As communicated by one father,

What was good about the conversations was that we all of us talked about how we experienced things, but in a new way . . . I didn’t really dare say exactly everything to the children, but in the conversations it emerged how I had felt and what I had experienced.

Although the parents had intended to talk about their emotions and anxieties regarding the breast cancer diagnosis, they said that the opportunity during treatment never arose. One of the mothers conveyed, “So, I think it’s good to get help talking about this in our everyday life. You tell yourself that today I’ll talk to the children, but it somehow doesn’t work out like that.” As the conversations were experienced as focused, the family members found it easier to communicate their emotions and their fears regarding the breast cancer diagnosis and its complications. The families asserted they felt safer and more relaxed as they gained insight into each other’s feelings and thoughts. “Children don’t want to talk about what they feel, you know, they are good at pretending that everything is alright, but now I got to know,” one father remarked. Furthermore, family members declared that they had gained extended knowledge of each other’s thoughts and feelings, which meant that they no longer needed to guess and interpret or protect. One son declared, “I’ve learned more about how mom thinks. Now I know. I’ve also been able to ask the questions I had and get them answered.” Moreover, the family members felt that by participating in the BFamHC conversations, they were not only encouraged to narrate their experiences and ask questions, but they were also invited to reflect on each other’s narratives at the same time as they heard new information in the responses to reflections that they had not heard previously.

Despite experiencing the conversations as safe and permissive and feeling that the presence of the conversational leader made it possible to communicate difficult emotions and experiences, family members revealed that they concealed certain thoughts in order not to transfer their own fear to another family member. One mother declared,

There are questions that we can discuss together, but there are also questions that should have been for me alone because I may express thoughts that transfer anxiety to my son; and I don’t want that, of course. So, I say or convey little in order to protect him, and I suppose that A (the son) does the same due to the fears he has in relation to me—we are, after all, mother and son.

Thus, some family members expressed a desire for both BFamHC individual and family conversations.

### Being Confirmed as an Individual and as a Family

By participating in the BFamHC conversations, the family members’ understanding of each other’s different experiences during the breast cancer trajectory was facilitated. The families felt seen and important, and they appreciated the interest they were shown as family members and as a family unit. Having a health care professional who wanted to listen to them and who had the requisite knowledge to be able to answer their questions made them feel confirmed and safe. One daughter commented, “It felt safe to have a person one could talk to, to ask questions if one had any, and to get the time to sit down together as a family.” By participating in the conversations, the families received more information and gained more knowledge; thus they felt more supported. One father asserted he appreciated “the fact that a person comes who has been there and who knows what’s what, who can ask questions and get a conversation going, but also give advice and support.”

The participation in BFamHC conversations allowed the families to understand that they could go through a difficult situation together. Consequently, they felt stronger as a family and as a unit. The families also felt that the extended knowledge gained during the conversations not only helped them to manage, but it also confirmed how they had dealt with the strain of the breast cancer diagnosis. The conversations also equipped them with tools for communicating emotions and difficult issues as a family. Moreover, the reflections made by the nurse during the conversations confirmed their feelings and thoughts as “normal” and that there are multiple ways of experiencing a situation. Overall, the families’ increased understanding of each other’s thoughts and feelings reinforced and confirmed the notion of each family collectively coping with a difficult situation. As articulated by one of the mothers, “Now that we’re faced with new challenges, these three conversations have given us a seal or confirmation that . . . well, that we’ll cope with this too.”

### Gaining an Unexpected Insight

Some family members claimed they were able to communicate about everything together. However, they believed that not all families had this ability. One mother remarked,

So, I suppose we haven’t had that need, but I suppose it’s important that it exists because there are people who are completely left to their own devices or who can’t talk to each other; and that would have been terrible.

For a number of families, accepting participation in the BFamHC conversations was primarily a means to help other families in the future. That said, participation in the BFamHC conversations provided a few families with insights into the actual communication patterns in their family. For others, the idea of being able to communicate about everything, including cancer, was negated.

The participation in these conversations made the families aware that their joint conversations as a family unit seldom involved emotions; rather, they tended to focus on practical issues. As expressed by one son, the participation in the BFamHC was an eye-opener:

In fact, I think one can begin to have doubts, and I think that’s quite good, being able to talk about and open up for how we actually solve things. You do that through stopping to think: okay, we’re very open, but do we really talk a lot to each other?

Adult children who were not living at home related having close contact with their family while emphasizing that they had rarely talked about their feelings or thoughts regarding their mother’s illness with the other family members. It also emerged that the parents had shared their experiences to a greater extent with each other, while the children rarely talked about what had happened. It was surprising for the families that the BFamHC had been valuable and beneficial for them. Furthermore, despite some families’ initial disbelief that they, as a family, would benefit from these conversations, they felt that their participation had shown them that every family might need support through the BFamHC. “If we hadn’t participated, we wouldn’t have been talking about it,” noted one of the mothers.

## Discussion

The interviews with the nine families to explore their experiences of participating in BFamHC following a breast cancer diagnosis enabled the research team to identify three key themes: bringing everything out in the open; being confirmed as an individual and as a family; and gaining an unexpected insight. Perhaps, the most significant finding in this study is that the family’s participation in the BFamHC was expressed as a positive experience, despite initial ambivalent feelings about participating. The family members received new insights into each other’s experiences through their participation in the BFamHC conversations. In addition, they found it helpful and even healing to be given the opportunity to tell their own story, as well to listen to the other family members’ illness narratives. By participating in the BFamHC, family members had an opportunity to hear their loved ones relate their emotional inner life, which had not been shared before. Furthermore, they felt that it was positive that they themselves dared to express feelings like fear. Through the opportunity to tell one’s story and disclose one’s experiences, individual family members may have attained an improved understanding of themselves (Ricoeur, 1992) and of their family members (Ricoeur, 1998), which might be regarded as a form of healing. Thus, participation in BFamHC and the narratives told in those conversations may affect the healing process and help family members to reflect on their present and future experiences.

The findings of this study suggest that families experienced their participation in BFamHC as an opportunity to share their thoughts and feelings with other family members. Similar findings have also been reported in previous studies using Family Health Conversations in the context of living as a family with various chronic diseases (Benzein et al., 2008, 2015; Clausson & Berg, 2008; Dieperink et al., 2017; Hollman Frisman et al., 2018; Holtslander, 2005; Houger Limacher & Wright, 2006; Östlund et al., 2016; Svavarsdottir & Sigurdardottir, 2013). These studies emphasize that the conversations strengthened feelings of togetherness through increased openness, sharing of feelings, as well as insights into each other’s experiences. Consequently, if the families in our study were to be interviewed again at a later time, this might be a valuable area to explore further. It would, furthermore, be of value to find out how long-lasting this feeling of togetherness might be.

Previous research has shown that family members often try to shield each other and experience difficulties in communicating their inner feelings following a breast cancer diagnosis (Coyne et al., 2012; Ginter & Radina, 2019; Holst-Hansson et al., 2017). Based on the findings of this study, BFamHC seem to be beneficial to families in providing an opportunity and helping them find ways of verbalizing their thoughts and feelings following a breast cancer diagnosis. Moreover, it is possible that their relational sharing may strengthen the family’s sense of being a unit and standing strong together throughout the entire illness trajectory.

Yet, another finding of interest is that all families expressed that the first BFamHC should have taken place earlier in the treatment trajectory. However, different opinions regarding the most appropriate time were expressed, which might be due to individual needs of the families in relation to the breast cancer diagnosis and different treatment regimens. Although routinely offered to every family, the timing for the initiation of conversations like BFamHC should be flexible and based on each family’s situation, needs, and preferences. Thus, conversations like BFamHC could be offered to families soon after the diagnosis, with multiple options for them to initiate the conversations later in the treatment trajectory. Inhestern and colleagues (2016) have also emphasized the need for flexible and accessible interventions and support programs offered as a routine care procedure. Furthermore, offering BFamHC early in the treatment trajectory is in line with statements made by the Institute of Medicine and National Research Council—who stress the significance of specific points along the cancer continuum, with particular attention given to the time for diagnosis and the end of treatment (Hewitt & Ganz, 2006). The time for diagnosis is identified as the beginning of an emotional roller-coaster ride, while the end of active treatment is often associated with a decline of support from health care professionals. Hence, there is a risk that unmet needs of support experienced by the families during cancer treatment (especially at the end of active treatment) might go undetected as the woman moves on to be a breast cancer survivor.

The families participating in this study experienced their home as a safe and optimal place for the BFamHC. The family home as a place that offers a sense of safety and control has also been found in studies by Årestedt et al. (2016), Bateson (1972), and Lindahl et al. (2011). Offering the families BFamHC in their home environment may help the family to experience themselves more as the unit they are. Moreover, it may balance the communication between the conversational leader (nurse) and the family, in the sense that the nurse is a guest in the family’s home (Ross & Johansen, 2002). Brief family nursing conversations (15 min or less) were originally developed for use as routine care in the clinical setting (Wright & Leahey, 2013). However, our families’ appreciation of having their home as a setting for the BFamHC might imply that they would be less inclined to participate if the conversations were to take place at a clinic or hospital. This is an important finding that needs to be further explored before concluding if BFamHC should be held in either a home or a clinical setting, or if the setting should be left for the family to decide.

In this study, the BFamHC lasted between 15 and 40 min, which is similar to the findings of Svavarsdottir et al. (2012), who evaluated short-term therapeutic conversations offering educational and emotional support to families following acute and chronic illness in children and adolescents. This finding indicates that the planned duration (15 min) might be insufficient time for a family to relate their narratives, for the conversational leader to pose reflective, interventive questions, or for the family members to answer questions. This raises the notion that there has to be certain flexibility and possibility to individualize the length of the conversation according to the needs and wishes of the families. The reason Wright and Leahey (2013) gave for limiting the conversations held with families to 15 min was the recurrent concerns raised by nurses about not having enough time to involve families in nursing care, which is an argument that is still valid (Dieperink et al., 2017; dos Santos Ribeiro Silva et al., 2013). Thus, when implementing BFamHC as routine care, the needs and wishes of the families may have to be balanced against the time the nurses have at their disposal, or there is a risk that the conversation may not be considered feasible.

The appeal from some of our participants for both individual conversations and family conversations, such as BFamHC, again shows the complexity in offering the right support. This further emphasizes that health care professionals should acknowledge the whole family and focus not only on the individual with breast cancer. There is a need for the perspectives of both family-related care and family-centered care along the cancer continuum. Family-related care focuses on the individual family member, that is, the person with the illness or other family members, whereas the family is regarded as the context. FSN, in contrast, focuses on the family as a unit from the perspective of several family members simultaneously (Benzein et al., 2017; Wright & Leahey, 2013). By regarding these two as complementing each other—that is, as family-focused care in the clinic—it is not a question of choosing the one or the other; rather, it is a matter of when to apply the family-related care and when to apply the FSN. Nurses and other health care professionals might have to take a flexible approach and assess the unique needs of both the woman and her family members to provide the most appropriate care for every woman and family following a breast cancer diagnosis.

The reflections offered by the conversational leader of the BFamHC were experienced as central to the positive experience of the participation in BFamHC, since the families felt that those reflections confirmed them as individuals and as a family. Hence, the reflections of the conversational leader or of the nurses in the clinical setting may help families begin to share their experiences following the breast cancer diagnosis and its subsequent treatment. Through their reflections, nurses also have the opportunity to acknowledge suffering due to an illness, use commendations, and challenge families’ constraining beliefs (Houger Limacher & Wright, 2006; Wright & Bell, 2009).

### Methodological Considerations

Throughout the whole research process, efforts were made to enhance trustworthiness according to the principles of Lincoln and Guba (1985), namely, credibility, dependability, confirmability, and transferability. As the study was based on an FSN approach, where the entire family’s experience is of interest, family interviews were conducted. The strength of family interviews is that a systemic perspective, with interactions between and within the family, came to light. The potential weakness of family interviews is the risk of family members not daring to reveal their opinions due to mutual consideration of each other’s feelings.

To increase the credibility of the study, all families were interviewed in the research conversations by the same interviewer (V.V.), who is a registered nurse, specialized in pediatrics, and with extensive experience of conducting research interviews with families. To further increase the credibility of the study, all four authors (A.H.H., V.V., E.I., and A.W.) participated in the analysis. The rational for including the first author (A.H.H.), who conducted the BFamHC in the role of conversational leader, was to broaden the awareness of all aspects of the research team’s pre-understanding. This decision may be considered controversial as it implies that the conversational leader in a sense explored her own effort. Thus, to maintain awareness of the research team’s own bias, the third (E.I.) and fourth (A.W.) authors were invited at a later stage of the analysis process to audit and confirm the findings. Dependability is demonstrated by detailed descriptions of all stages in the analysis process.

A possible limitation of the study is the small sample of families that participated and that they were only from one part of Sweden; this needs to be considered in terms of the transferability of the results. Nevertheless, the findings of these elucidating interviews should contribute to a richer understanding of families’ experiences of participating in BFamHC when a family member is diagnosed with breast cancer.

## Conclusion

The findings of this study confirm the importance of BFamHC and benefits of therapeutic conversations between nurses and families which, according to the International Family Nursing Association Position Statements for Generalist Competencies for Family Nursing Practice (2015) and Advanced Practice Competencies for Family Nursing (2017), contribute and facilitate movement toward family health. In addition, the findings support the usefulness of offering brief therapeutic conversations, such as BFamHC, with families following the diagnosis of breast cancer. However, it is our belief that further research is needed to establish when in time BFamHC may be initiated to optimize its outcome for families following a breast cancer diagnosis. We also believe that further research is needed to establish whether BFamHC can be used for family members following other cancer diagnoses.
